# Epigenetic Changes Affecting the Development of Hepatocellular Carcinoma

**DOI:** 10.3390/cancers13164237

**Published:** 2021-08-23

**Authors:** Ewa Wolinska, Maciej Skrzypczak

**Affiliations:** 1 Department of Pathology, Medical University of Warsaw, 02-091 Warszawa, Poland; 2 IInd Department of Gynecology, Lublin Medical University, 20-059 Lublin, Poland; skrzypczakmk8@gmail.com

**Keywords:** hepatocellular carcinoma, epigenetics, DNA methylation, histone modification

## Abstract

**Simple Summary:**

Hepatocellular carcinoma is a life-threatening disease. Despite many efforts to understand the exact pathogenesis and the signaling pathways involved in its formation, treatment remains unsatisfactory. Currently, an important function in the development of neoplastic diseases and treatment effects is attributed to changes taking place at the epigenetic level. Epigenetic studies revealed modified methylation patterns in HCC, dysfunction of enzymes engaged in the DNA methylation process, the aberrant function of non-coding RNAs, and a set of histone modifications that influence gene expression. The aim of this review is to summarize the current knowledge on the role of epigenetics in the formation of hepatocellular carcinoma.

**Abstract:**

Hepatocellular carcinoma (HCC) remains a serious oncologic issue with still a dismal prognosis. So far, no key molecular mechanism that underlies its pathogenesis has been identified. Recently, by specific molecular approaches, many genetic and epigenetic changes arising during HCC pathogenesis were detected. Epigenetic studies revealed modified methylation patterns in HCC tumors, dysfunction of enzymes engaged in the DNA methylation process, and a set of histone modifications that influence gene expression. HCC cells are also influenced by the disrupted function of non-coding RNAs, such as micro RNAs and long non-coding RNAs. Moreover, a role of liver cancer stem cells in HCC development is becoming evident. The reversibility of epigenetic changes offers the possibility of influencing them and regulating their undesirable effects. All these data can be used not only to identify new therapeutic targets but also to predict treatment response. This review focuses on epigenetic changes in hepatocellular carcinoma and their possible implications in HCC therapy.

## 1. Introduction

Hepatocellular carcinoma (HCC) is the third leading cause of cancer-related deaths worldwide [1]. It represents the most common form of liver cancer, responsible for over 90% of primary liver cancers. Current treatment options, such as hepatic resection, radio-frequency ablation, chemoembolization, or liver transplantation, are relatively effective in the early stages of HCC, when patients retain liver functional reserve. However, many patients are diagnosed at a late stage and, therefore, are not eligible for such a treatment. A high percentage of patients die because of metastases or recurrence. Alternative or palliative treatment is very limited due to resistance to chemotherapy or radiotherapy. Thus, the average survival rate of the treated patient is approximately two years [2]. 

## 2. HCC Etiology and Prevalence

The most important risk factor of HCC is liver cirrhosis, present in up to 90% of patients [3]. This condition usually develops as a result of infection with hepatitis B (HBV) or hepatitis C (HCV) virus, aflatoxin B uptake, or alcohol consumption. In some cases, the etiology is associated with cirrhosis that results from a coexisting liver disease, such as non-alcoholic fatty liver disease (NAFLD), or some hereditary diseases, such as hemochromatosis [4].

The HCC prevalence is more common in Eastern Asia and sub-Saharan Africa; however, in Western countries (northern Europe, North America, and Australia), HCC incidence is rising, which is associated not only with hepatitis or high alcohol intake but also with the growing percentage of obese people in these populations [5]. Meta-analysis data suggest that a high body mass index increases the relative risk of liver cancer [6]. An additional factor affecting the incidence of HCC is gender. In fact, HCC occurs more frequently in men, with a male-to-female ratio ranging from 2:1 to 4:1 [4]. This difference was assigned to steroid sex hormones and their nuclear receptors [7]. Although significant differences in nuclear androgen and estrogen receptors functioning have been found in men and women with HCC, no significant clinical implications have been identified. The high technologies currently used to study changes occurring during cancer pathogenesis at the cellular and molecular levels have brought much important information regarding HCC. In this review, we summarize the most crucial dysfunctions of signaling pathways and factors that lead to and facilitate the development of this most common primary liver cancer.

**Figure 1:**
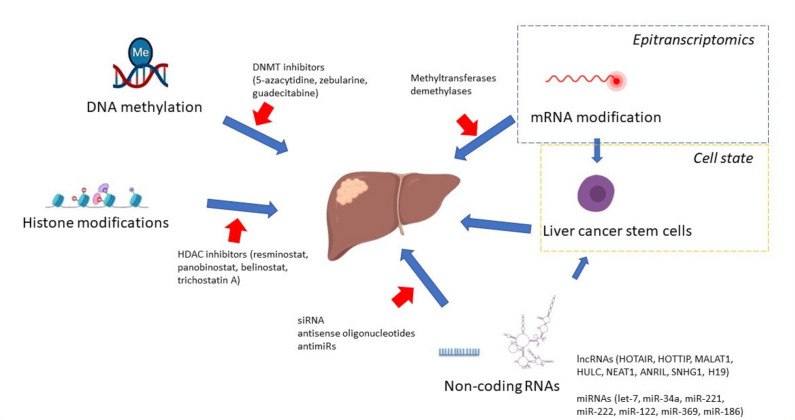
Epigenetic processes implicated in hepatocellular carcinoma development. Dnmt DNA methyltransferase, HDAC histone deacetylase, siRNA small interfering RNA, lncRNAs long noncoding RNAs, and miRNAs micro RNAs.

So far, no single key signaling pathway has been found to represent the molecular switch leading to the development of HCC, but numerous signaling pathways modifications implicated in other neoplasms are also important in this condition. Most of them affect cancer cell proliferation, invasiveness, migration, resistance to apoptosis, and changes in the microenvironment that facilitate tumor survival. As recent reports show, changes in the behavior of tumor cells occur at many levels of cell functioning (Figure 1).

## 3. DNA Methylation Pattern in HCC

One of the key epigenetic processes found to promote tumorigenesis is an aberrant DNA methylation pattern. Regions that are usually affected by aberrant hypermethylation are promoter sequences of genes responsible for cell cycle regulation, apoptosis, DNA repair, metabolism of carcinogens, and angiogenesis (Table 1) [8]. Genome-wide analyses revealed frequently decreased hypomethylation of cancer genomes when compared with non-cancerous liver tissue [9,10,11]. A comprehensive genome analysis also revealed that within such hypomethylated regions, somatic mutations may occur with a higher frequency [11]. In HCC, hypomethylation was found to activate such proto-oncogenes as c-Jun and c-myc [12]. In addition, this type of gene regulation may promote carcinogenesis by influencing mitotic recombination, which may lead to increased genomic instability [12]. The same authors have shown that global hypomethylation in liver cancer is independent of various etiological factors.

On the contrary, hypermethylation may disturb the function of another group of genes. One of the signaling pathways affected by hypermethylation is the WNT/β-catenin signaling. In over 80% of human HCC, the tumor suppressor gene adenomatous polyposis coli (*APC*) is inactivated by hypermethylation [13]. This causes aberrant accumulation of β-catenin in cell nuclei and activation of its oncogenic properties. Another protein whose gene is aberrantly methylated in HCC is one of the cell cycle regulators, p16INK4A. Multiple studies revealed frequent (in up to 85% of the cases studied) methylation of the *p16INK4A* gene [14]. The absence of p16INK4A, a potent cell checkpoint regulator, promotes aberrant cell proliferation. Since it can be detected not only in tissues but also in the serum of patients, it can serve as a potentially useful marker for early HCC diagnosis.

The other important signaling pathway affected during HCC development by epigenetic disturbances is the methylation of the tumor suppressor genes that regulate Ras, i.e., *RASSF1A* and *NORE1A* [15]. Uncontrolled Ras activation in HCC influences cell growth and differentiation.

Another group of genes that are aberrantly methylated and may promote tumorigenesis are those encoding the DNA repair system. In HCC, promoter methylation of the mismatch repair system genes (*MMR*) is commonly detected, and the inactivation of their proteins is observed [16]. The most important enzymes of this family are hMLH1, hMSH2, and hMSH3. [16]. Abnormal methylation of their genes may occur at a frequency of up to 75% [16,43]. Since this type of aberration is also found in cirrhotic liver, surrounding a tumor mass, this aberration may represent a rather early step of hepatocarcinogenesis [44].

Research based on bioinformatic analysis has the advantage of integrating many data sources. In such a study by Liang et al., methylation-regulated differentially expressed genes (DEGs) of potential prognostic value in HCC were presented [17]. A total of 9 upregulated and 72 downregulated genes were identified in this study. Among them, 4—*CTF1* (cardiotrophin-1), *FZD8*, *PDK4* (pyruvate dehydrogenase kinase 4), and *ZNF334*—were found to be negatively associated with overall survival. In addition, the methylation status of *CDF1* and *PDK4* was identified as an independent prognostic factor. In a study of Fan et al., 19 hypomethylated and 14 hypermethylated genes were identified, among which 6 key hub genes (*MAD2L1*, *CDC20*, *CCNB1*, *CCND1*, *AR*, and *ESR1*) were specified [18]. These aberrantly methylated genes were associated with the cell cycle process and with p53 and MAPK signaling. Further analysis revealed that *MAD2L1, CDC20,* and *CCNB1* play an oncogenic role, whereas *CCND1*, *AR,* and *ESR1* are associated with favorable patient survival. Qiu et al. have found that the CpG methylation signature can be useful in predicting recurrence in early-stage HCC [45].

An integrated bioinformatic analysis may also help to identify diagnostic biomarkers to differentiate between different types of liver cancer. For instance, Bai et al. described methylation sites typical for HCC but not cholangiocarcinoma [46]. Future research may allow the estimation of the prognostic and diagnostic value of integrated bioinformatic data from HCC cells and tissues.

## 4. Function of DNA Methyltransferases in HCC

The reaction of transferring a methyl moiety to the 5-carbon of cytosine is catalyzed by a class of DNA methyltransferases (Dnmts), of which mainly three enzymes play a role in cancer development and progression: Dnmt1, Dnmt3A, and Dnmt3B. Dnmt1 functions mainly during cell division and is called maintenance Dnmt while Dnmt3a and Dnmt3b methylate DNA de novo during cellular differentiation [47]. Studies on HCC have revealed that overexpression of the genes encoding methyltransferase family members is associated with poor patient survival [48]. Increased expression of *DNMT3A* and *DNMT3b* was associated with poorer differentiation and shorter metastasis-free survival of HCC patients [49]. Pre-clinical studies showed that Dnmt inhibitors, such as 5-azacytidine or zebularine, exert an antitumor effect on HCC cells in vitro. This effect was induced by both an epigenetic reversion of the malignancy-associated phenotype and an efficient re-sensitization to apoptosis-inducing substances, such as TRAIL [50,51]. In HCC cell lines, 5-azacytidine and the histone methyltransferase EZH2 inhibitor 3-deazaneplanocin A (DZNep) enhance the efficacy of immunotherapy for HCC by the activation of transcriptionally repressed genes [52].

## 5. Histone Modifications

Histones may undergo posttranslational regulation via many processes, such as acetylation, deacetylation, methylation, ubiquitylation, or sumoylation of lysine residues. The acetylation and deacetylation processes are regulated by two classes of enzymes: histone acetyltransferases (HATs) and histone deacetylases (HDACs) [53]. HATs catalyze the transfer of an acetyl group to the histone side chains of lysine and thus relax the interaction between the histone and chromatin. HDACs, in turn, reverse lysine acetylation and thus stabilize chromatin structure, making it less available to transcription factors. Histone modification can accompany cancer pathogenesis, possibly due to the influence on the expression of oncogenes and tumor suppressor genes or to the alteration of chromatin structure, enabling/disabling the access of transcription factors to the gene regulatory sites. HDAC1 and HDAC2 are related to HCC development; however, each of them exerts this role in a different way. *HDAC1* expression was correlated with moderately and poorly differentiated tumors [54], whereas *HDAC2* expression was identified as an independent negative prognostic factor of survival [55]. HDAC2 is involved in the epigenetic regulation of cell cycle, apoptosis, and differentiation and was found to be commonly upregulated in HCC [19]. Small interfering RNA (siRNA)-mediated silencing of *HDAC2* inhibited HCC growth in vitro (accompanied by the deregulation of HDAC-regulated genes, such as *p27*, *p53*, *BCL-2*, or *PPAR-γ*) and in vivo in mouse xenograft models [19]. HDAC3 regulates the cell cycle and proliferation. Its downregulation increased the *p21WAF1*/*cip1* expression and hence induced G1 phase arrest [20]. Additionally, HDAC3 plays a role in STAT3-dependent cell proliferation in liver regeneration and cancer [21]. *HDAC3* silencing induces a number of effects on STAT3; it impairs its transition from acetylation to phosphorylation and inhibits its nuclear translocation. It also decreases growth of HCC xenografts. Studies on HDAC3 function in HCC pathogenesis revealed also its dependence on tumor necrosis factor receptor-associated factor 6 (TRAF6). TRAF6 ubiquitinates HDAC3, which results in the blockage of HDAC3-mediated histone H3 and oncoprotein c-Myc deacetylation, leading to *c-Myc* mRNA expression and enhanced c-Myc protein stability and, consequently, enhanced liver tumorigenesis [22]. HDAC3 function is also related to double-strand breaks repair via targeting the H3K9ac/H3K9me3 transition [23]. HDAC3 ablation interrupted the deacetylation and consequent trimethylation of H3K9 (H3K9me3), the first step in double-strand break repair, and led to the accumulation of damaged DNA.

HDAC3 and HDAC1 were also shown to jointly regulate cell migration, epithelial–mesenchymal transition (EMT), and tumor metastasis. As Hu et al. showed, both HDACs suppressed the expression of the gene encoding the zinc-finger transcription factor Snail2 through deacetylation of H3K56 and H3K4, which triggered the repression of Snail2-mediated EMT [24].

HDAC8 was also found to be significantly upregulated in both HCC cell lines and tumor tissues when compared with human normal hepatocytes and corresponding non-tumor tissues. In addition, HDAC8 inhibition significantly inhibited hepatoma cell proliferation and transformation activity via the upregulation of RB1 in vitro and in vivo [25]. In a study by Fan et al., it was reported that the mRNA and protein levels of HDAC5 were upregulated in human HCC tissues and cells, whereas the downregulation of HDAC5 inhibited cell proliferation in HepG2, Hep3B, and Huh7 cell lines and tumor growth in a xenograft model [26]. In addition, the suppression of *HDAC5* resulted in cell apoptosis and induced G1-phase cell cycle arrest in HCC cells by altering the levels of pro- and antiapoptotic proteins (p53, bax, bcl-2, cyto C, and caspase 3) and cell cycle regulators (cyclin D1 and CDK2/4/6). The *HDAC9* overexpression, relative to normal liver, was also documented in HCC tissue [56]. *HDAC9* expression level is an independent negative predictor of poor prognosis and survival. HDAC9 also functions in the epithelial–mesenchymal transition process and acts as a regulator of the differentiation and acquisition of stemness properties in HCC cells. A study of Kanki et al. revealed that the level of *HDAC9* mRNA positively correlated with the markers of mesenchymal phenotype and stemness and negatively correlated with hepatic differentiation markers [27]. The inhibition of HDAC9 in undifferentiated HCC cells decreased its ability of anchorage-independent cell growth and self-renewal. In general, *HDAC* expression seems to be increased in HCC, but there appear to be differences among patients and among particular HDACs. As most pharmacological HDACs inhibitors non-selectively block various HDACs, a thorough understanding of the expression and function of specific HDACs is key to effectively targeting them in HCC therapy.

## 6. Non-Coding RNAs

Non-coding RNAs, such as miRNAs, have now a well-defined position as developmental regulators in numerous diseases, including cancer [57]. Several miRNAs were found to be involved in liver cancer pathogenesis, including let-7, miR-34a, miR-221, miR-222, and miR-122. In a study on HCC tumor samples and their corresponding nontumorous counterparts, the expression profiles of miRNAs were assessed. It was found that, among several miRNAs studied, miR-221/222 was the most upregulated in tumor samples. The upregulation of miR-221/222 led to enhanced cell growth in vitro by targeting the CDK inhibitor p27 [28]. Further analysis led to the identification of its target, DDIT4, a regulator of mTOR kinase, whose downregulation may result in HCC pathogenesis. Additional functional studies on a mouse model of liver cancer demonstrated that miR-221 overexpression stimulated the growth of tumorigenic murine hepatic progenitor cells [28]. Another study showed that miR-369, which targets zinc finger E-box-binding homeobox 1, is downregulated in liver fibrosis and liver cancer tissues, and can predict a poor prognosis in HCC patients [29]. Additionally, when miR-369 expression was restored, proliferation and metastasis of HCC cells in vitro and in vivo were inhibited. Numerous other miRNAs were related to HCC cells proliferation, apoptosis, as well as chemoresistance: miR-3174 by targeting FOXO1, miR-383 by targeting IL-17 via STAT3 signaling pathway, and miR-361-5p by targeting CXCR6, VEGFA, or MAP3K9 [30,31,32,58,59]. Another important property of miRNA molecules is their effect on cancer stem cells. miR-186 knockdown facilitates liver CSCs self-renewal and tumorigenesis [33]. miR-186 is a newly discovered miRNA that directly targets protein tyrosine phosphatase non-receptor type 11 (PTPN11) by binding to its 3′UTR. PTPN11 is upregulated in liver CSCs and promotes liver CSC expansion [34]. It is required for miR-186-mediated liver CSC expansion and chemoresistance of HCC cells. The expression of miR-186 was lower in cisplatin-resistant HCC cell lines when compared with non-malignant cells, as well as in cisplatin-resistant patient-derived xenograft tissues [33].

The malfunction of specific miRNAs can be targeted using synthetic inhibitors, such as antisense oligonucleotides or AntimiRs. For instance, oligonucleotides targeting miR-221 reduced tumor cell proliferation and increased markers of apoptosis and cell cycle arrest in an orthotopic xenograft mouse model [60]. An miRNA cocktail encapsulating miR-199a/b-3p mimics (miR199) and anti-miR-10b was found to effectively inhibit HCC cells proliferation and tumor growth by targeting mTOR, PAK4, RHOC, and epithelial–mesenchymal transition pathways both in vitro and in vivo [61]. Moreover, miR-423-5p treatment sensitized HCC cells to sorafenib therapy [32]. Stiuso et al. found the secretory miR-423-5p upregulated both in vitro and in vivo after sorafenib treatment, and its increase correlated with response to therapy. In 75% of patients in which such an increase in secretory miR423-5p was found, partial remission or stable disease after six months from the beginning of therapy was observed [62]. miR-122 expression was reduced in a subset of HCC patients, including hepatitis B virus (HBV)-positive patients with highly invasive and metastatic cancer [35]. As previous studies showed, mice with depleted miR-122, systemically or only in the hepatocytes, developed spontaneous HCC after one year of life [63]. This implies that miR-122 plays a role as a tumor suppressor in HCC, and its restoration may inhibit tumor growth. Such studies in animal models have already shown promising results [35].

An increasing body of evidence points out the involvement of long non-coding RNAs (lncRNAs) in HCC pathogenesis [36]. lncRNAs make up a broad group of RNA transcripts that can change gene expression via various mechanisms: by recruiting transcription factors to chromatin regions or preventing their binding or by affecting other factors that can change the structure of chromatin [36,64]. lncRNAs represent a very potent group of molecules that can act by binding both RNA (including miRNAs) and proteins and thus can change gene expression at the transcriptional and protein levels [36]. In a large study based on The Cancer Genome Atlas, it was revealed that dysregulation of lncRNA is specific to the cancer type [65]. Several lncRNAs are related to HCC development by affecting cell proliferation, motility, metastatic ability, and angiogenesis. Such molecules have a positive correlation with patients’ clinicopathological parameters but also affect patients’ sensitivity to chemotherapeutics. An example of an lncRNA with a well-established role in HCC is the HOX transcript antisense intergenic RNA (HOTAIR), implicated in HCC proliferation, regulation of pluripotency, metastasis, and sensitivity to doxorubicin and cisplatin [36]. Another molecule from this group whose expression affects survival, tumor grade, and poor prognosis, is HOTTIP [37]. Interestingly, HOTTIP expression was found to be progressively upregulated in the transition from cirrhotic liver to HCC [66]. Another lncRNA whose expression correlates with advanced tumor stages and reduced overall survival of HCC patients is MALAT1 (metastasis-associated lung adenocarcinoma transcript 1) [67]. In addition, its expression levels correlated with increased risk of HCC recurrence after liver transplantation [68]. Another interesting study showed that the nuclear genome-encoded lncRNA MALAT1 functions as a critical epigenetic player in the regulation of mitochondrial metabolism in HCC cells [38]. The authors demonstrated that MALAT1 is enriched in the mitochondria, affects both their function and their number, and its knockdown induces alterations in the structure, transcriptome, and function of mitochondria. MALT1 dysregulation together with another lncRNA, HULC (highly upregulated in liver cancer), promotes the growth of liver cancer stem cells [39]. On the other hand, HULC overexpression promotes the progression of HCC cells and inhibits the chemosensitivity to the anti-cancer drug oxaliplatin [40]. The mechanism of its action involves an increase in cell proliferation, protective autophagy, and inhibition of apoptosis. HULC knockdown increased the chemosensitivity to oxaliplatin through the repression of cell growth and the acceleration apoptosis in HCC cells. Further analysis confirmed its mode of action through the regulation of the miR-383-5p/vesicle-associated membrane protein-2 axis, the miR-377-5p/HIF-1α pathway, and the miR-134-5p/FOXM1 axis [41,42]. Emerging evidence provides new insights on the role of other lncRNAs in HCC development, such as NEAT1, ANRIL, SNHG1, or H19 [36,69]. Since lncRNAs expression, similarly to that of miRNAs, can be regulated by silencing through siRNA or antisense oligonucleotides, they are a possible target for anticancer therapy. However, it needs to be noted that the therapeutic use of RNAi presents several obstacles, including incomplete suppression of target genes, efficient in vivo delivery to target cells, and nonspecific immune responses [70]. 

## 7. N6-Methyladenosine mRNA Modification

Emerging evidence highlights the role of RNA methylation in cancer development [71]. N6-methyladenosine (m6A) is the most abundant form of mRNA modification. Under physiological conditions, it plays a role in various aspects of RNA metabolism, such as nuclear export, translation, decay, and alternative splicing [72]. The correct functioning of m6A is controlled by two classes of enzymes, methyltransferases and demethylases. Once their balance is disturbed, m6A function is abnormal, which can lead to numerous effects including tumorigenesis. As was demonstrated by Shen et al., m6A plays an essential role in the molecular pathogenesis of HCC [71]. These authors showed m6A modifications to be related to the infiltration of immune cells into the HCC microenvironment and the regulation of the anti-tumor immune response.

## 8. HCC Risk Factors and Epigenetics

HBV, a major HCC etiologic factor, is able to induce epigenetic alterations in the host via multiple mechanisms. Among viral proteins, HBx was found to play a significant role as an epigenetic regulator [73]. HBx can upregulate members of the DNA methyltransferase family, such as Dnmt1, Dnmt3A1, and Dnmt3A2, and selectively promote the regional hypermethylation of specific tumor suppressor genes (i.e., *RASSF1A*, *GSTP1*, and *CDKN2B*). Additionally, HBx expression correlates with genomic hypomethylation, as documented both in vitro and in vivo, and with the regional hypermethylation of insulin-like growth factor binding protein-3 (IGFBP-3). It is noteworthy that this effect on IGFBP-3 appears to be an early event during HBx-mediated hepatocarcinogenesis, as it is observed even in HBx-positive nontumor tissues adjacent to the tumor tissues [73].

A number of tumor suppressor genes have also been reported to be hypermethylated in HCV-infected HCC (*RASAL1, EGLN3, CSMD1, CDKN2A, BCORL1, SFRP1, ZNF382, RUNX3, LOX, RB1,* and *P73*) [74]. The process of epithelial-to-mesenchymal transition is epigenetically regulated by HCV through the inactivation of WNT/β-catenin signaling [75]. In a study of Zhou et al., it was demonstrated that HCV core protein silences secreted frizzled-related protein 1 (SFRP1), an extracellular signaling molecule that antagonizes Wnt signaling [76]. SFRP1 expression can be restored using a DNA methylation inhibitor or its combination with a histone deacetylase inhibitor (HDACI). The expression of *DNMT1*, *DNMT3A*, and *DNMT3B* was upregulated in HCV-infected liver cancer; however, this was found only in some patients [77]. Hepatitis C virus core protein downregulates E-cadherin expression via the activation of DNA methyltransferases 1 and 3b [78]. Other epigenetic effects of HCV include hepatocyte apoptosis regulation [79] and cell cycle regulation via promoter hypermethylation of p16 [80] or via growth arrest and DNA damage (Gadd45) gene family [81]. Another study showed that HCV infection induced genome-wide epigenetic changes in histone modifications, which altered cellular signaling pathways in HCC [82]. Interestingly, such changes persisted after curing the viral infection. Since these changes can be reversed by epigenetic modulators, further research may provide an opportunity to prevent HCC progression.

Non-alcoholic fatty liver disease (NAFLD) and alcoholic liver disease (ALD) are common HCC etiologic factors. Published data indicate that particular epigenetic changes can be associated with these HCC etiologic factors. NAFLD has been connected with miR-21, miR-34a, and miR-182 upregulation and miR-122 downregulation [83]. miR-122 downregulation affects lipogenesis in cellular models. Moreover, in mouse models, hepatic deletion of miR-122 induces spontaneous development of NASH via increased lipogenesis and impaired lipid secretion, which subsequently progresses to HCC [84]. miRNAs related to ALD include miR-155, miR-34a, miR-122, miR-212, and miR-21 [85]. As Ambade et al. showed, alcoholic hepatitis accelerates early hepatobiliary cancer by increasing stemness and miR-122-mediated HIF-1α activation [86]. Increased hepatic methylation of the peroxisome proliferator-activated receptor γ coactivator 1, involved in mitochondrial function, has been associated with NAFLD [87]. An epigenome-wide analysis performed on NAFLD patients’ data identified seven CpG sites whose DNA methylation was associated with fibrosis [88]. In another genome-wide study, 208 CpG islands were found to be differentially methylated when comparing normal and cirrhotic liver [89]. The level of global DNA methylation was lower in NAFLD patients and tended to decrease with an increase in hepatic inflammation and fibrosis grade [90].

## 9. Liver Cancer Stem Cells

Within liver tumor tissue, cells that possess stem cell properties were identified [91]. Cancer stem cells (CSCs) have the ability to self-renew and produce a subset of cells differentiated in different directions [92]. CSCs can recapitulate the tumor when implemented in immunocompetent mouse models. Early studies were focused on identification of reliable markers of such a cell population. In liver cancer cell lines, CD133 expression can be used as a marker of a CSC-enriched cell population [93]. Further studies on HCC tumor samples confirmed that CD133-positive cells can indeed be found within a cancer cell population in different amounts, and their presence negatively correlates with clinical outcome [94]. A CD133-positive cell population is also more resistant to both chemo- and radiotherapy and has a higher ability to form metastases [95,96]. Other markers of CSC-enriched cells are CD44, CD24, EpCAM, CD90, CD13, and OV6 [97].

Recently, a new liver progenitor-specific gene, RNA-binding RALY-like (RALYL) protein, was identified [98]. RALYL belongs to a heterogeneous nuclear ribonucleoprotein (hnRNP) family of RNA-binding proteins that are involved in transcriptional and post-transcriptional regulation. It was found that RARYL increases the stemness of HCC by affecting the mRNA stability of transforming growth factor beta (TGF-β) through the decrease in N6-methyladenosine modification [98]. The upregulation of other stemness-related markers, such as CD133, was also associated with RARYL overexpression.

Since HCC self-renewal, chemoresistance, and metastasis formation are strongly affected by cancer stem cells—their function and regulation—these cells constitute a possible target for therapy. Effective eradication of such cancer cell population could considerably improve the efficacy of HCC treatment.

## 10. Targeted Therapies

Although numerous molecular changes were identified in HCC, their use as therapeutic targets in the clinics and therapy is still poor. Over the last decade, only sorafenib, a multikinase inhibitor, was approved for systemic HCC therapy. More recently, new multikinase inhibitors, such as regorafenib, Lenvatinib, and cabozantinib, were introduced into the treatment of patients non-responsive to sorafenib therapy [99]. Regorafenib, the most extensively studied so far, is a multikinase inhibitor with a higher pharmacological potency than sorafenib [100]. Interestingly, some genetic polymorphisms of genes encoding enzymes involved in metabolic pathways as well as miRNAs were found to correlate with the outcome of the regorafenib therapy [99]. Genetic variability in the genes *CYP3A4* and *UGT1A9* was reported to have a predictive value for regorafenib hepatotoxicity. Cabozantinib is another multikinase inhibitor that can modulate different cellular pathways, including angiogenesis, as well as oncogenic pathways in HCC, implicated in tumor progression and metastasis. This drug received Food and Drug Administration approval in 2018 for the treatment of advanced sorafenib-resistant HCC [101]. Lenvatinib is an oral, small-molecule tyrosine kinase inhibitor, which targets multiple tyrosine kinase receptors and is offered to patients with advanced unresectable HCC. Although in clinical trials, it showed better outcomes than sorafenib did, its treatment efficacy is often unsatisfactory due to adverse effects [102]. According to the recent guidelines from the American society of clinical oncology, the combined treatment with atezolizumab–bevacizumab is recommended as a first-line therapy for advanced HCC [103]. Other drugs, such as ramucirumab, pembrolizumab, or nivolumab, are also considered.

The reversibility of epigenetic changes is currently broadly investigated as a possible cancer therapeutic option. For instance, HDAC inhibitors are already approved for the treatment of cutaneous T cell lymphoma and peripheral T cell lymphoma [104,105]. Clinical trials with the HDAC inhibitor resminostat combined with sorafenib in patients with HCC proved their safety and showed early signs of efficacy [106]. Additionally, resminostat, as was documented in HCC cell lines, changes the mesenchymal phenotype of cells towards a more epithelial, and thus less invasive, phenotype, which may contribute to sensitization to sorafenib-induced apoptosis [107]. Additional leverage of this drug combination is its influence on the platelet-mediated pro-tumoral effect in HCC [108]. It has been documented that platelet factors antagonize the action of kinase inhibitors, such as sorafenib. In a phase I/II clinical trial conducted in patients with advanced HCC previously untreated with systemic chemotherapy, the combination of resminostat and sorafenib resulted in longer overall survival than sorafenib monotherapy in patients with a higher baseline platelet count [109]. Another inhibitor, Panobinostat, has shown encouraging results in combination with sorafenib in preclinical studies, but this effect has not yet been confirmed in clinical trials [110]. Belinostat, a pan-HDACI studied in unresectable HCC patients, demonstrated tumor stabilization and was generally well tolerated in patients, despite its limited efficacy [111]. Epigenetic drugs have not only antitumor activities but also can modify antitumor immunity [112]. Preclinical studies in murine HCC models confirmed enhanced anti-tumor activity when belinostat was used in combination with immune checkpoint inhibitors [113]. As Llopize et al. showed, belinostat combined with simultaneous blockade by two inhibitors, CTLA-4 and PD-1, led to complete tumor rejection in a subcutaneous murine HCC model. Such encouraging results provide a rationale for testing belinostat in combination with checkpoint inhibitors to enhance their therapeutic activity in patients with HCC.

Another HDAC-1 inhibitor with immunomodulating properties is trichostatin A [114]. In vitro studies demonstrated the enhanced killing of HCC cells by NK cells, whereas in vivo, trichostatin A reduced tumor cell growth in an NK cell-dependent manner. Given its additional abilities to inhibit the growth of cancer cells and induce apoptosis, this inhibitor is a promising agent in HCC therapy.

The second class of epigenetics-targeted drugs for HCC are DNA methylation inhibitors, DnmtI. First-generation DnmtI, including 5-azacytidine and 5-aza-2’-deoxycytidine (decitabine), showed efficacy and were approved for the treatment of hematologic neoplasms. However, due to their short half-life after administration, their use is limited to solid tumors [115].

Guadecitabine, a second-generation DNA methyltransferase inhibitor, showed greater antiproliferative effect in preclinical studies. Both in vitro and in vivo studies on HCC cell lines and mouse xenograft models confirmed its inhibitory properties on cell growth as well as delayed tumor growth [116]. An additional combination with oxaliplatin revealed improved efficacy [117]. A clinical study combining guadecitabine and durvalumab (an anti-PD-L1 monoclonal antibody) to treat solid tumors, including HCC, is currently ongoing (NCT03257761). As shown by growing evidence from preclinical studies, therapy aimed at HCC epigenetic targets in combination with chemotherapeutic agents or immunotherapy has a chance to bring a breakthrough in HCC treatment. 

## 11. Conclusions

Taken together, the above results indicate that a comprehensive knowledge of the molecular changes occurring during HCC pathogenesis may not only have an impact on the overall survival of patients but also promote the optimal design of targeted therapies. Such information has also an important predictive value on other aspects of therapy, facilitating the assessment of possible drug toxicities or the identification of biomarkers associated with drug response.

## Figures and Tables

**Table 1:** Summary of key epigenetic changes in HCC and their consequences.

Epigenetic Changes	Mechanism Affected	Reference
DNA methylation		
Hypomethylation	Protooncogene c-Jun and c-myc activation	[12]
	Mitotic recombination/genomic instability	[12]
Hypermethylation	WNT/β-catenin signaling activation	[13]
	APC inactivation	[13]
	p16INK4A activation	[14]
	RASSF1A and NORE1A activation	[15]
	Mismatch repair system genes (hMLH1, hMSH2, and hMSH3) inactivation	[16]
	Cardiotrophin-1 (CTF1), FZD8, pyruvate dehydrogenase kinase 4 (PDK4), and ZNF334 activity	[17]
	MAD2L1, CDC20, CCNB1, CCND1, AR, and ESR1	[18]
	p53 and MAPK signaling regulation	[18]
Histone modification		
Upregulated HDAC2	Dysregulation of cell cycle, apoptosis, and differentiation via p27, p53, BCL-2, or PPAR γ	[19]
Downregulated HDAC3	An increase in p21WAF1/cip1 expression; G1-phase arrest	[20]
Downregulated HDAC3	STAT3-dependent cell proliferation	[21]
Downregulated HDAC3	c-Myc protein synthesis and stability	[22]
Downregulated HDAC3	Defective double-strand breaks repair	[23]
HDAC3 and HDAC1	Cell migration, epithelial-mesenchymal transition (EMT), and tumor metastasis regulation	[24]
Upregulated HDAC8	Downregulation of RB1	[25]
Upregulated HDAC5	Increased cell proliferation	[26]
Downregulation of HDAC5	Cell apoptosis via antiapoptotic proteins (p53, bax, bcl-2, cyto C, and caspase 3), G1-phase cell cycle arrest via cell cycle regulators (cyclin D1 and CDK2/4/6)	[26]
Upregulated HDAC9	Epithelial–mesenchymal transition process activation; cellular stemness properties regulation	[27]
Non-coding RNAs		
miR-221/222	Enhanced cell growth via p27 regulation mTOR kinase regulation	[28]
miR-369	Zinc finger E-box binding homeobox 1 regulation	[29]
miR-3174	FOXO1 regulation	[30]
miR-383	IL-17 via STAT3 signaling pathway regulation	[31]
miR-361-5p	CXCR6, VEGFA, or MAP3K9 regulation	[32]
miR-186	CSCs self-renewal	[33]
miR-186	Protein tyrosine phosphatase non-receptor type 11 regulation	[34]
miR-122	Tumor growth regulation	[35]
HOTAIR	Proliferation, regulation of pluripotency, metastasis, and sensitivity to chemotherapeutics	[36]
HOTTIP	Survival, tumor grade, and prognosis	[37]
MALAT1	Regulation of mitochondrial metabolism	[38]
HULC	Growth of liver cancer stem cells	[39]
	Chemosensitivity of anti-cancer drug oxaliplatin inhibition	[40]
	Regulation of miR-383-5p/vesicle-associated membrane protein-2 pathway; miR-377-5p/HIF-1α pathway and miR-134-5p/FOXM1 pathway	[41,42]
